# Intrinsically Disordered Energy Landscapes

**DOI:** 10.1038/srep10386

**Published:** 2015-05-22

**Authors:** Yassmine Chebaro, Andrew J. Ballard, Debayan Chakraborty, David J. Wales

**Affiliations:** 1 Department of Chemistry, University of Cambridge, Lensfield Road, Cambridge CB2 1EW

## Abstract

Analysis of an intrinsically disordered protein (IDP) reveals an underlying multifunnel structure for the energy landscape. We suggest that such ‘intrinsically disordered’ landscapes, with a number of very different competing low-energy structures, are likely to characterise IDPs, and provide a useful way to address their properties. In particular, IDPs are present in many cellular protein interaction networks, and several questions arise regarding how they bind to partners. Are conformations resembling the bound structure selected for binding, or does further folding occur on binding the partner in a induced-fit fashion? We focus on the p53 upregulated modulator of apoptosis (PUMA) protein, which adopts an 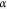-helical conformation when bound to its partner, and is involved in the activation of apoptosis. Recent experimental evidence shows that folding is not necessary for binding, and supports an induced-fit mechanism. Using a variety of computational approaches we deduce the molecular mechanism behind the instability of the PUMA peptide as a helix in isolation. We find significant barriers between partially folded states and the helix. Our results show that the favoured conformations are molten-globule like, stabilised by charged and hydrophobic contacts, with structures resembling the bound state relatively unpopulated in equilibrium.

Intrinsically disordered proteins (IDPs) defy the textbook structure-function paradigm, according to which a protein folds into a single and unique 3D structure to accomplish its physiological function. With their flexibility and inherent plasticity, IDPs play an important role in protein-protein interactions^1^ in many cellular processes, such as signal transduction and gene expression^2^, and therefore represent an important challenge in structural biology^3^. The presence of intrinsically disordered regions in cancer-associated proteins has highlighted their implication in human diseases^4^, as exemplified by p53 5 and HPV^6^. Amyloid aggregation and pathological assembly of IDPs also characterise neurodegenerative diseases, for example, the A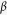 peptide in Alzheimer’s^7^.

Although unsuitable for high resolution X-ray crystallography, increasing efforts have been invested in the last decade in many other experimental techniques to characterise the conformational ensemble of IDPs, such as NMR^8^,^9^, SAXS^5^,^10^, and mass spectrometry^11^.

As major components of protein-protein interaction networks, the disordered nature of an IDP is advantageous in many ways^12^, including fast association with alternative partners, highlighting the prevalence of IDPs in cell signalling pathways. From a structural point of view, disordered proteins are noteworthy in undergoing a disorder-to-order transition upon binding to a partner, referred to as the ‘coupled folding and binding mechanism’^13^,^14^. This binding mechanism rules out a lock-and-key scenario, highlighting a key question: does binding occur through conformational selection or induced-fit? In the first case the binding partner selects a conformation closely related to the IDP-bound conformation, although not necessarily most populated in the unbound ensemble. In the latter case, the binding partner induces structure and folding of the disordered protein upon contact. These two scenarios are not mutually exclusive, but could be system dependent, and molecular simulation has proved to be a crucial tool in this challenging field. Monte Carlo simulations have recently shown how two different IDPs bind to the same partner in a similar manner^15^. Multicanonical molecular dynamics simulations were performed to enhance sampling, and identified a cooperative induced-fit and conformational selection procedure for the binding of a 15-residue IDP^16^. A sophisticated multistate G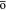-model was recently applied to show that the NCBD disordered protein binds two different partners, mainly through an induced-fit recognition mechanism, although the authors do not exclude an alternative conformational selection pathway for binding^17^.

Here we focus on the PUMA protein (p53 upregulated modulator of apoptosis), belonging to the Bcl-2 homology 3 (BH3)-only subclass of proteins, and leading to the activation of apoptosis when bound to its partner the antiapoptotic protein Mcl-1^18^. The region of PUMA that binds Mcl-1 is called the BH3 region; this motif is intrinsically disordered and adopts a helical structure when bound to its globular folded partner^19^. Recent biophysical studies have demonstrated that PUMA associates rapidly and tightly to its partner Mcl-1^20^. Upon introducing proline substitutions in the peptide sequence^21^ (thus breaking helical structure), association rate constants with Mcl-1 are mainly unaffected, suggesting that particular residual helical structure does not seem to be essential for binding. Using rapid-mixing stopped flow, Rogers and coworkers^22^ have recently shown that neither folding nor specific interactions are required for association, suggesting that conformational selection for binding is unlikely. Thus, it seems that the IDP PUMA binds its partner in an induced-fit fashion. From a structural perspective, why then isn’t the alpha-helical conformation of PUMA stable in isolation?

To answer this question, we employ an array of computational methods, ranging from molecular dynamics to creation of a kinetic transition network^23^,^24^,^25^ using geometry optimisation. Our results show that indeed isolated PUMA is not stable as a contiguous 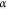-helix and the conformational ensemble is populated by structures with mainly low to medium helicity content, in agreement with experiment^20^. Visualizing the energy landscape of PUMA in isolation reveals a frustrated^26^,^27^ multifunneled structure with no dominant energy minimum and molten globule-like structures at the bottom of the funnels. Most importantly, we show that (i) the barriers to folding for an 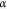-helix from the most populated conformations are large, (ii) unfolding is orders of magnitude faster than folding, and (iii) the driving forces to unfolding of the contiguous helix are mainly electrostatic in nature, but not necessarily residue-specific. These results help to explain the instability of this IDP when isolated, as well as the induced fit mechanism for binding determined by experiment^22^.

Diverse electrostatic and hydrophobic interactions are formed within the partially folded structures of PUMA, leading to the presence of the multiple partially folded conformations, and form the basis for the intrinsically disordered behaviour and the corresponding ‘intrinsically disordered energy landscape’, which seems likely to characterise such proteins. This structure provides a useful way to address emergent observable properties from the energy landscape perspective.

## Results

### The 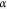 -helical conformation of the PUMA peptide unfolds in isolation

**Figure 1:**
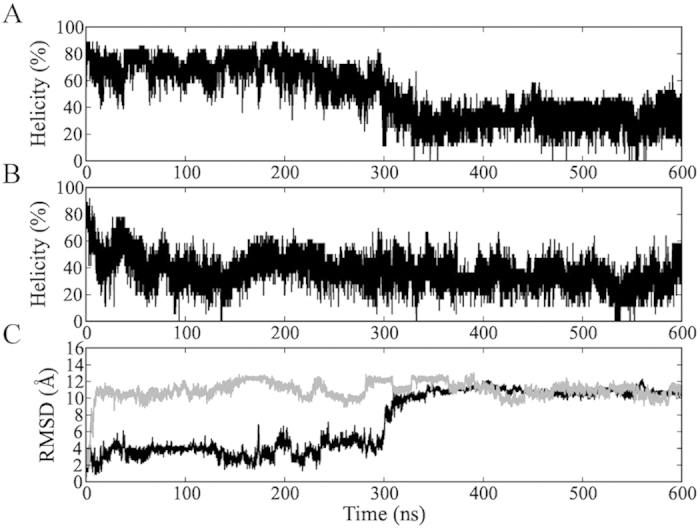
(**A, B**) The percentage of residual helicity and (**C**) backbone RMSD with respect to the minimised 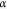-helix conformation of PUMA, where the MD starting points are the helix at 280 (**A** and black lines in **C**) and 300 K (**B** and grey lines in **C**).

To obtain some initial insight into the 32-residue PUMA peptide when isolated, we performed 600 ns molecular dynamics simulations starting from the contiguous helical structure at 280 and 300 K in explicit solvent (Fig. 1). At 280 K, the percentage of residual helicity reaches 40% after 15 ns and fluctuates between 40 and 80% over a time scale of 200 ns. At 300 K, the helicity rapidly decreases to 35% after 20 ns. Over the last 100 ns of the MD simulations, the average helicity values are 32 and 30% at 280 and 300 K, respectively (Fig. 1A,B). To monitor the similarity to the contiguous helical PUMA, we calculated the backbone root mean square deviation (RMSD) and our results show a rapid increase to more than 3 Å after only 17 ns at 280 K. At 600 ns, we observe relatively large values for the RMSD, ranging from 10 to 12 Å in all simulations, following the trends of helical percentage (Fig. 1C). In each case, the structures become more collapsed, and their radius of gyration decreases from about 16 Å to around 10 Å (Supplementary Figure 1A). This increase in the compaction of the isolated protein is evident from calculating distances between two residues located at the N- and C-terminal regions, which decrease from high (over 35 Å) to low (under 5 Å) values (Supplementary Figure 1B). From these results it is clear that the helix is not stable by itself, consistent with the induced-fit scenario identified experimentally^20^,^21^,^22^.

Conventional molecular dynamics simulations are prone to kinetic trapping, and to enhance the conformational sampling of the helical PUMA peptide in isolation, we performed 140 ns of replica exchange molecular dynamics (REMD)^28^ using 20 temperature replicas ranging from 223.7 to 650 K in implicit solvent, resulting in a total simulation time of 2.8 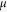
s. The first 30 ns of the simulation were excluded from the analysis to allow for equilibration. REMD simulations were deemed to have converged when the heat capacity and residual helicity values calculated in the time intervals 30 to 85 and 85 to 140 ns showed similar behaviour (Supplementary Figure 2). A heat capacity peak appears at 313 K, where the residual helicity is about 24%.

**Figure 2:**
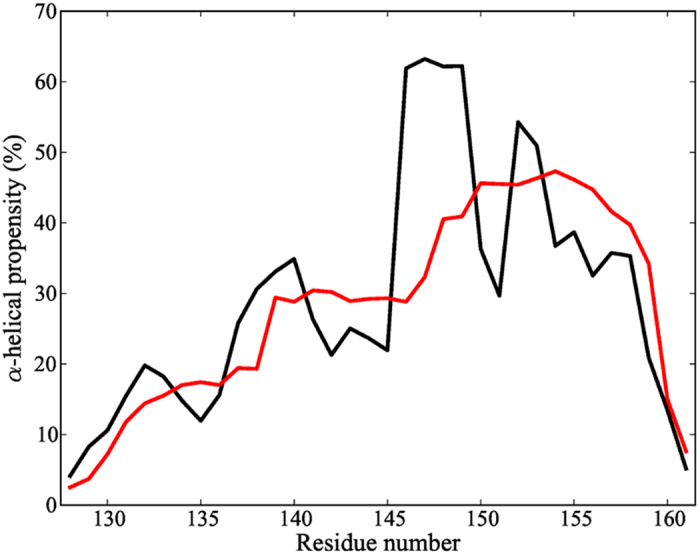
Helical propensities at the residue level for the PUMA sequence calculated from REMD simulations (black lines) and from the AGADIR^29^ helical propensity predictor (red lines).

At 280 K the average helicity in our simulations is 27.8% and that calculated with the AGADIR predictor^29^, used by Rogers and coworkers to design point mutations in the PUMA sequence^21^, is 28%. These values are consistent with CD experiments suggesting about 20% helicity at 298 K^20^. Calibrating simulation temperatures and experimental ones is a difficult task, especially when force fields are used with an implicit solvent representation. The temperatures used for this comparison are not exactly the same, but we believe that 280 K approximates best the experimental room temperature, in view of the heat capacity curve obtained from our simulations (Supplementary Figure 2) suggesting that at 298 K the system is too close to the melting regime. Although slightly over-estimating the helicity with respect to experiment, the force field reproduces the experimental range of values with no significant differences. These results are supported by the comparison of different force fields using NMR scalar coupling data, which suggest that the AMBER representation we have used has the best agreement with experimental data among those tested^30^. We calculated the helical propensities at a residue level at 280 K for the last 110 ns of the REMD simulations and compared the values obtained with AGADIR^29^ (Fig. 2). Overall, the two profiles agree in the general trends of helicity, where the highest helical content is present mainly in the C-terminal region. The largest discrepancies appear at positions A150 and Q151, but the 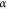-helical percentages for these residues are still elevated (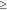
30%).

**Figure 3:**
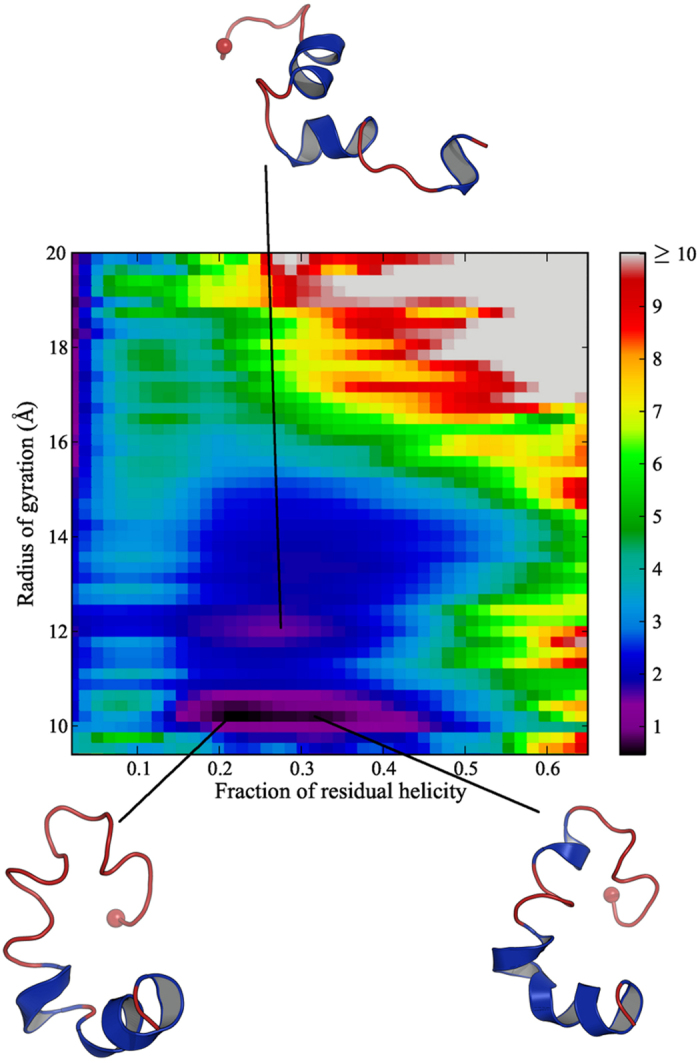
Free energy surface at 280 K for the PUMA peptide in terms of fraction of residual helicity and radius of gyration. The free energy scale in kcal/mol is shown on the right. Typical conformations for the minima are represented, where the C_α_ of the N-terminal residue Val128 is indicated by a sphere.

A free energy surface for the PUMA peptide was constructed at 280 K, using as order parameters the fraction of residual helicity and the radius of gyration (Fig. 3). The lowest free energy minimum is rather broad and corresponds to a range of fractional helicity between 0.2 and 0.4, with a radius of gyration of about 10 Å. The other low-lying free energy minimum corresponds to a more extended structure, with a radius of gyration of around 12 Å, but covering the same range of helicity. It is important to note that regions with residual helicity greater than 0.6 exhibit relatively high free energies, and correspond to values around 15 to 16 Å for the radius of gyration (close to the contiguous helix), confirming that the helical structure of the PUMA peptide is unfavourable.

### Visualising the conformational ensemble of the IDP using discrete path sampling

**Figure 4:**
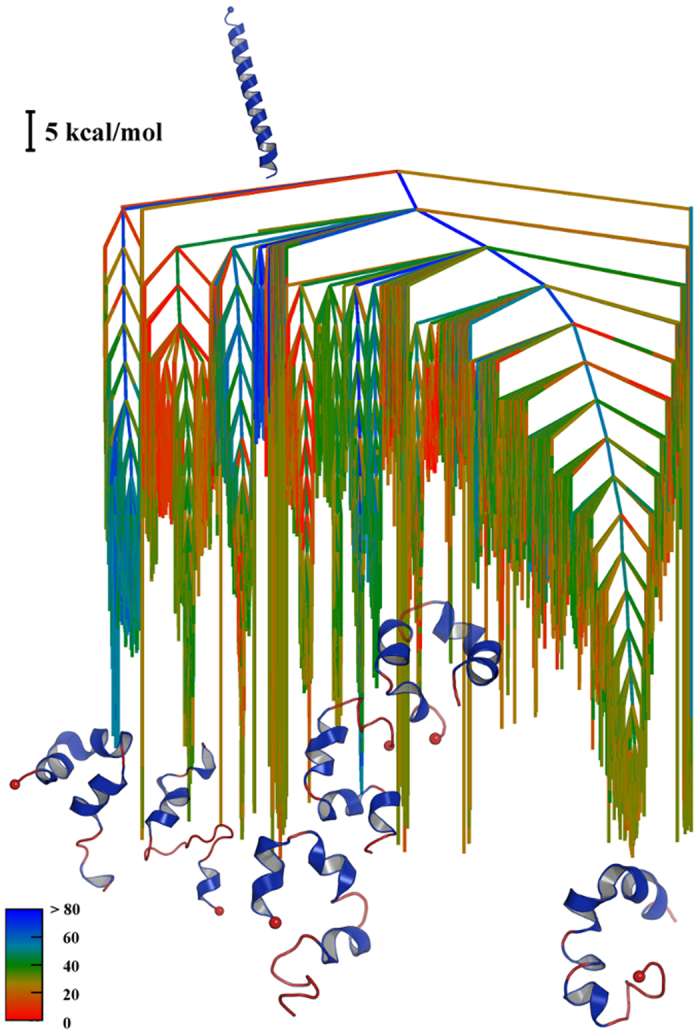
Potential energy disconnectivity graph constructed from the most populated structures obtained in the REMD simulations at 280 K. The colouring of the branches corresponds to the 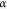-helical percentage calculated for each minimum in the database, as defined in the key.

We used the discrete path sampling (DPS) approach^31^ to analyse the underlying potential energy landscape for PUMA. The DPS technique is based on geometry optimisation and efficiently produces stationary point databases corresponding to a kinetic transition network^23^,^24^,^25^. To extract relevant partially folded PUMA structures, clustering analysis was performed on the trajectory at 280 K, based on dihedral angles, which groups structures that display similar secondary structure content. We considered the ten most populated structures at 280 K, which in total account for 45% of the analysed simulation time frames, as starting points for building a connected database, together with the helical structure of PUMA. The combined stationary point database from DPS calculations is visualised using a potential energy disconnectivity graph^32^^,^^33^ in Fig. 4 and consists of a total of 205,677 minima and 191,392 transition states. The colouring in this figure corresponds to the percentage of helicity for each minimum in the database. At the bottom of the funnels, the helicity percentages are between 20 and 30%; the lowest and highest helicity values are not favourable, and such structures are located at branches near the top of the graph. Frustration^26^,^27^ in the disconnectivity graph corresponds to low-lying morphologies separated by high barriers, and illustrates the diversity of competing conformations, with no single dominant structure. The corresponding free energy disconnectivity graph is illustrated in Supplementary Figure 3, and it displays the same structure as the potential energy landscape for the relevant temperature range. The observed difference between the 2D free energy surface calculated from the REMD simulations and the disconnectivity graph obtained using the DPS technique occurs because some partially folded states are incorrectly lumped together on projection to obtain a low-dimensional representation. Such projections of configuration space do not generally preserve the kinetics, often producing artifically smooth surfaces that do not reflect the barriers properly, thus masking the complexity of the landscape^25^,^34^,^35^,^36^. In contrast, a kinetic transition network can faithfully represent the barriers^25^. Here we note that for complex biomolecular pathways a suitable progress coordinate may not exist. Such conformational transitions are therefore ideal targets for DPS, which only requires specification of product and reactant states.

The multifunnel structure seems likely to be associated with the intrinsically disordered nature of the protein. Hence the disconnectivity graph representation provides a visual explanation for the induced fit mechanism of binding. In particular, it is clear that the contiguous helix conformation of PUMA (i) does not occupy a low energy funnel and (ii) the barriers to unfolding are much lower than the ones to fold into the helix. In fact, the funnel corresponding to the contiguous helix is relatively narrow and underpopulated with respect to the other funnels. The most populated structure at 280 K (12.4%) corresponds to the largest funnel in terms of local minima, although it competes with low energy funnels related to the other most populated structures.

Such multifunnel energy landscapes have been extensively studied for atomic and molecular clusters^37^, where they provide benchmarks for global optimisation, enhanced sampling schemes to address broken ergodicity^38^,^39^,^40^,^41^, and rare event dynamics^31^,^42^,^43^, corresponding to changes of morphology. The competing funnels lead to multiple relaxation time scales and features in the heat capacity profile^25^, and correspond to glassy behaviour in systems with an exponentially large number of low-lying amorphous states^44^. Consistent with the present results, a multifunneled energy landscape has previously been characterised for an amyloid peptide derived from the disordered domain of the yeast prion protein Sup35. As for PUMA, this system exhibits competition between alternative conformations, which are 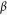-sheet structures for the amyloid^45^. Hence, we suggest that IDPs are likely to correspond to intrinsically disordered, multifunneled energy landscapes. We propose to test this hypothesis for other IDPs in future work.

We calculated the phenomenological rate constants for the unfolding and folding from the contiguous helix (conformation A) and the most populated structure at 280 K (conformation B), using graph transformation^46^. The comparison of these rates indicate that folding is slow with respect to unfolding, indeed the estimate for 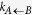 is 0.27 × 10^−20^ s^−1^ (probably a weak lower bound) whereas 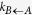 is over 10.5 s^−1^. We also calculated the rates for conversion of the helix to the other most populated structures (Table 1). These transitions are also several orders of magnitude faster than the predicted folding rates of each of these structures to the helix. The numerical values cannot be compared directly with binding rate constants, since the partner MCL-1 is absent in these calculations, and it is more appropriate to interpret the relative rather than the absolute rates. The particularly slow folding rate obtained for the helical conformation supports the lack of evidence for a conformational selection for binding^22^ and suggests that the presence of the partner may accelerate this transition.

Rate constants between the initial partially folded minima used to build the energy landscape of PUMA were calculated using the same procedure (Supplementary Table S1). Apart from the first two conformations, which are similar in structure, (Cα-RMSD of 1.7 Å), the transitions are relatively slow between the partially helical states. Although no experimental results for these interconversion rates between partially folded states for the free PUMA protein have been reported so far, it is known that conformational fluctuations in IDPs can be slowed by the presence of residual structure^47^. Indeed, experimental studies on an analogue of the BPTI show slow interconversion rates between two partially folded conformations with different degrees of order^48^. NMR relaxation experiments for the compact molten globule NCBD domain of transcription factor CBP reported the presence of slow conformational fluctuations in this IDP^49^. A theoretical study on an archetypal IDP sequence revealed slow interconversion between distinct conformations of the peptide in water^50^.

**Figure 5:**
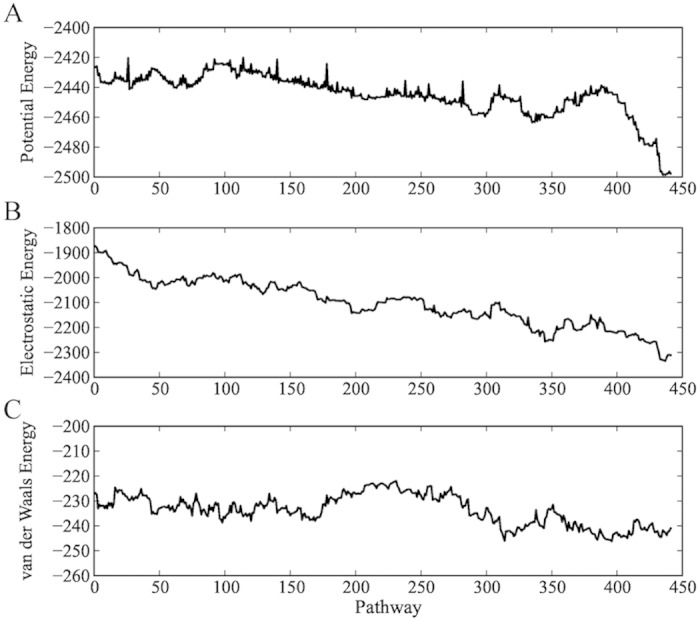
Energies in kcal/mol along the fastest path for the transition between the contiguous helical conformation of PUMA and the partially folded structure corresponding to the most populated conformation at 280 K. The potential energy (**A**) of each starting point is decomposed into (**B**) electrostatic and (**C**) van der Waals components.

To understand the molecular mechanism of unfolding, we calculated the electrostatic and van der Waals components of the potential energy for the successive minimum-transition state-minimum stationary points in the discrete path^31^ leading from the helix to the most populated structure at 280 K (Fig. 5). This pathway is mainly downhill in energy, and the most important forces driving the unfolding are electrostatic (Fig. 5B), with a gain of approximately 400 kcal/mol on forming a more globular structure. The corresponding change in the van der Waals energy is negligible in comparison, with only about 10 kcal/mol differece between the two end points (Fig. 5C). Hence it seems that electrostatics drive the unfolding and determine the instability of the contiguous helical form.

### Structural characterisation of the partially folded ensemble

**Figure 6:**
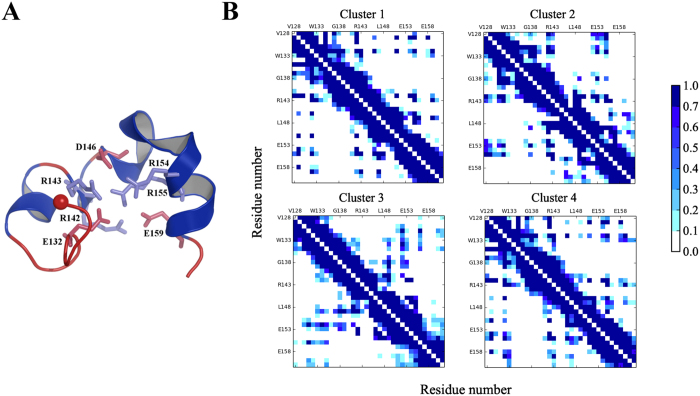
(**A**) Most populated structure at 280 K, where the hydrogen bonds with an occurrence greater than 50% of the simulation time are included for the analysis. Residues implicated in the bonds are represented by sticks, the N-terminal C_α_ of the PUMA sequence is indicated by a sphere. (**B**) Contact maps calculated for the structures for the four most populated clusters.

To provide a more detailed understanding of the electrostatic characteristics of PUMA at the residue level, we first analysed the hydrogen-bond network, defined using a distance cutoff of 3 Å and a maximum deviation from a linear angle of 40°. All the hydrogen bonds present for more than 40% of the simulation time considered for the analysis are associated with side chains of charged residues. The four most prevalent hydrogen bonds involve residues D146 with R154 and R155, R142 and E132, and R143 and E159. Looking at the positions of these charged residues in the structure of the most populated cluster (Fig. 6A), we see that they are located around the center of the U-shaped PUMA conformation.

We then performed a structural analysis of the PUMA protein and calculated contact maps for the structures in the four most populated clusters (Fig. 6B). Interactions between charged and hydrophobic residues are observed. For example, the strongest hydrophobic contacts involve I137, G138 and A139 with L141, and M144 with L141. Significant interactions between charged residues occur for E132-R135, R143-D146 and R156-E158. These hydrophobic and charged interactions are also the most important ones observed in the contact map calculated for all the structures observed at 280 K (Supplementary Figure 4), where the number of the most populated hydrophobic and charged contacts lie within the same sequence ranges of 8 and 11 contacts, respectively. However, in the most populated cluster, it seems that the charged interactions are more prevalent than the hydrophobic ones, with 24 most populated compared to 10. Although some contacts are similar, different interaction patterns are observed for each cluster (Fig. 6B). This result could explain the diversity in the PUMA structures and hence the absence of one dominant single fold for the protein in the absence of binding partners, as suggested when looking at the population of each cluster at the same temperature (Table 1) and by the multifunneled character of the energy landscape.

Mutations of charged residues to alanine (for instance R143) do not destabilize the binding of PUMA to MCL-1^21^. Although we cannot infer any details of the binding process, we can suggest explanations for this observation. It is conceivable that the disruption of just one charged residue does not perturb the electrostatic network much (Fig. 6A), as other charged residues may replace the missing contributions.

To characterise the effect of this substitution, we mutated one of the residues, R143, involved in the electrostatic network into an alanine in the structure of the most populated cluster at 280 K. The mutated structure was considered as a starting point for basin-hopping global optimisation using the GMIN program^51^. Here, we used rotation of side chain groups to explore the configuration space and hence investigate the effects of this mutation on the electrostatic network of the wild-type geometry. Overall, the resulting structures display the same pattern of charged interactions. The RMSD calculated using carbon atoms of the side chains is 0.6 and 1 Å for the six residues discussed previously (E132, R142, D146, R154, R155, E159) in the R143A mutant. This result again reflects the non-specific character of the electrostatic network formed within the PUMA protein.

Analysis of amino acid composition for IDPs has indeed shown that they typically contain numerous charged residues^52^,^53^, and the alternative electrostatic networks that can be formed in addition to hydrophobic contacts may contribute to the variety of molten-globule-like conformations observed in the PUMA protein when unbound, and hence its intrinsically disordered nature. Hydrophobic contacts could explain the overall preservation of helicity when varying the ionic strength, as determined experimentally^20^. This globule-like phase in the conformational space of IDPs has been linked to the net charge per residue^54^ and the charge patterning in the sequence^55^. In strong polyampholytic IDPs, with an elevated fraction of charged residues as for PUMA, the distribution of the charges in the sequence will influence the conformational properties of the protein. Das and Pappu^55^ have quantified this effect using a patterning variable termed 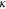, which ranges from 0 to 1, for a very symmetric distribution of charges and thus hairpin-like structures due to long-range electrostatic interactions between residues. For PUMA, 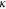 is about 0.18 according to the webserver CIDER^56^, which correlates well with the partially folded states identified in our simulations.

## Discussion

Experimental evidence regarding the binding of the intrinsically disorered protein PUMA to its partner, involved in subsequent activation of apoptosis in cells, suggests an induced fit mechanism^21^,^22^. These recent results show that no specific residual helicity or particular fold seem to be required for binding. In the present contribution, we have used a combination of computational methods to understand the behaviour in the absence of the binding partner to explain the induced fit mechanism. Our results show that the contiguous helical conformation of the protein is unstable, and the protein conformation ensemble is mostly molten globule with low to medium residual helicity, in agreement with CD results^20^. Most importantly, our aim was to understand the molecular mechanism that drives the destabilisation of the helix in isolation.

Visualisation of the corresponding energy landscape reveals a multifunnel structure, with a number of alternative low-lying morphologies separated by high energy barriers. The funnel representing the helical structure is clearly unfavourable with respect to the partially folded structures, explaining why conformational selection for this protein is unlikely and has not been observed experimentally^22^. The calculated rate constants corresponding to the transition from the PUMA helix to partially folded molten-globule-like states indicate a fast unfolding relative to folding, governed mainly by electrostatic forces, while the reverse process is predicted to be much slower. We have also shown that interactions between both charged and hydrophobic residues contribute to the stability of the partially folded states, and thus to the structure of the landscape and its emergent properties.

Mutations of single charged residues into alanine do not perturb either association or dissociation of PUMA to its partner MCL-1^21^, and we believe the reason is that the electrostatic network involves several charged amino acids, so mutation of one of these components to alanine can be compensated by another nearby charged residue.

We suggest that the multiple funnel structure is likely to characterise the landscapes of intrinsically disordered proteins, and this hypothesis will be tested in future work. The characterisation of such intrinsically disordered energy landscapes may provide a general approach to understand and calculate the properties of such systems.

## Methods

### Structure preparation

To compare our results with the studies of Rogers and coworkers^20^, we chose to work with the sequence 128-161 of the PUMA protein (Uniprot Q99ML1). The 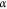-helical structure of the peptide was built using the PyMol software and is very similar to the NMR structure of the peptide (residues 130-156) in the bound state with MCL-1 (pdb id: 2ROC)^19^, with an RMSD of 1 Å. Molecular dynamics simulations were carried out using the AMBER99SB^57^ force field via the AMBER12 package^58^. NMR structural and relaxation data of the A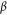 peptide were obtained using this force field in good agreement with experimental data, justifying its use for IDPs^59^. The N-terminus and the C-terminus were acetylated and amidated respectively. To eliminate steric clashes between atoms, a minimisation consisting of 500 steepest-descent steps was performed, followed by 500 steps of conjugate gradient, until the root mean square (RMS) gradient of the potential energy reached 10^−4^ kcal mol^−1^Å^−1^.

### Dynamics Simulations in Explicit Solvent

The initial helical structure was solvated with TIP3P^60^ water molecules in a box of dimension 330 nm^3^, with periodic boundary conditions applied for all molecular dynamics (MD) simulations. The SHAKE algorithm^61^ was applied to constrain bonds involving hydrogen atoms, and a time step of 2 fs was applied. The temperatures used in the MD simulations were 280 and 300 K, and a Langevin thermostat^62^ was used with a collision frequency of 2 ps^−1^. An initial minimisation with position restraints using a force constant of 500 kcal mol^−1^Å^−2^ on the PUMA protein was performed using 500 and 1,500 steepest-descent and conjugate gradient steps, respectively, in order to locally equilibrate the water and ions. Another minimisation was performed without any restraints on the protein for 1,000 steepest-descent steps followed by 3,000 conjugate gradient steps. Both minimisations were continued until the RMS gradient of the potential energy reached 10^−4^ kcal mol^−1^Å^−1^. Next, the solvent was heated to the required temperature for 20 ps with moderate restraints on the protein using a force constant of 10 kcal mol^−1^ Å^−2^. To allow the solvent density to equilibrate, a 500 ps NPT simulation was then performed with isotropic position scaling to a reference pressure of 1 bar. Finally, the production simulations using NVT conditions were run for a total of 600 ns at each temperature.

### Replica Exchange Molecular Dynamics

Replica exchange molecular dynamics (REMD)^28^ simulations were performed using the relaxed conformation of PUMA with 20 replicas at temperatures ranging from 223.7 to 650 K. The generalised Born model was used for an implicit solvent representation^63^. Temperatures were controlled using a Langevin thermostat with a collision frequency of 2.0 ps^−1^. The maximum interatomic distance for computing the effective Born radii was set to 25 Å, and no truncation was applied to the nonbonded interactions. Each simulation was equilibrated for 400 ps to reach the selected temperature for the REMD simulations. The production run consisted of a total of 140 ns for each replica, resulting in a total simulation time of 2.8 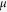
s. Exchanges were attempted every ps leading to an acceptance ratio of approximately 22%. The analysis was performed using the ptraj tools in the AMBER program, excluding the first 30 ns of the simulation for convergence purposes. The multistate Bennett acceptance ratio method (MBAR) of Shirts and Chodera^64^, a binless-equivalent to the weighted histogram analysis method^65^, was used to analyse the REMD trajectories. Clustering of the trajectories was performed on dihedral angles using the cluster tool in the MMTSB toolset^66^ with an angle cutoff of 40^º^. Secondary structure analysis was performed using the DSSP implementation in the AMBER trajectory analysis tools^67^.

### Exploring the potential energy landscape with basin-hopping

Exploration of the potential energy landscape was performed largely via basin-hopping^68^,^51^, an efficient strategy for global optimisation. The method consists of trial perturbations in configuration space, each followed by local energy minimisations, resulting in a set of configurational minima on the potential energy landscape. The acceptance criteria for a given trial move are determined by the energy difference between minima, as well as a user-specified temperature parameter. Because detailed balance is not required, the method affords great flexibility in choosing step sizes and temperature parameters that optimise sampling. As a result, basin-hopping can rapidly explore the potential energy landscape.

Basin-hopping was performed for the PUMA mutation R143A with 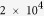 steps. The trial moves consisted of Cartesian displacements, pivot moves (i.e. rotations of the amino acid chain about a backbone dihedral angle), and rotations of R-group segments about various R-group bonds.

### Discrete path sampling

After clustering the REMD trajectory at the desired temperature, pathways connecting the 10 most populated clusters of PUMA were calculated, along with the contiguous helical conformation of the protein. Transition state candidates were produced as initial guesses using the doubly-nudged^69^ elastic band algorithm^70^. These candidates were tightly converged using a hybrid eigenvector-following approach^71^. Local minima were obtained using a modified limited-memory Broyden-Fletcher-Golgfarb-Shano (L-BFGS) algorithm^72^. Once an initial connected path between the chosen end points was found, the stationary point database was grown by addition of all minima and transition states identified in successive connection attempts for pairs of minima already existing in the database^73^. This approach, discrete path sampling (DPS)^31^, is implemented in the program PATHSAMPLE, which organises parallel connection attempts using the OPTIM program.

The rate constant between the two selected end points can be expressed as the sum over all discrete paths between product and reactant states^31^,^74^. The discrete paths that make the largest contribution to this rate constant were calculated using the Dijkstra algorithm, and the highest potential energy barriers on this path were identified. The procedure SHORTCUT BARRIER^45^ implemented in PATHSAMPLE was used to refine the database, until the rate constants converged to within an order of magnitude. Artificial kinetic traps were eliminated using the UNTRAP procedure^45^, which attempts to connect minima close in distance to the product set but separated by high barriers. The resulting database consisted of 205,677 minima and 191,392 transition states and was visualised using disconnectivity graphs^32^^,^^33^. The database of minima obtained from the DPS method was used to calculate free energies using the harmonic superposition approximation^75^. Phenomenological two-state rate constants, denoted k_*AB*_ and k_*BA*_ for the transitions from *B* to *A* and *A* to *B* respectively, were calculated using the graph transformation approach^74^^,^^76^ from the complete database after regrouping self-consistently structures that were separated by free energy barriers below a certain threshold^77^ (3 kcal/mol in this work).

## Author Contributions

Y.C., A.J.B. and D.C. performed the simulations and analyzed the data. Y.C., A.J.B., D.C. and D.J.W. discussed the results and wrote the manuscript.

## Additional Information

**How to cite this article**: Chebaro, Y. *et al*. Intrinsically Disordered Energy Landscapes. *Sci. Rep.* **5**, 10386; doi: 10.1038/srep10386 (2015).

## Supplementary Material

Supplementary Information

## Figures and Tables

**Table 1:** Estimated rate contants (in s^−1^) at 280 K for the unfolding and folding from the contiguous 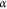 -helical conformation of the PUMA peptide to each of the 10 most populated structures from the REMD simulations, and their respective populations. The values for folding should be considered as lower bounds.
