# Supplementary material for: Intrinsically Disordered Energy Landscapes
Source: Sci Rep. 2015 May 22;5:10386. doi: 10.1038/srep10386 (PMC4441119; doi:10.1038/srep10386)
Supplement: Supplementary Information [file srep10386-s1.pdf]

Intrinsically Disordered Energy Landscapes

Yassmine Chebaro, Andrew J. Ballard, Debayan Chakraborty, and David J. Wales*

Department of Chemistry, University of Cambridge, Lensfield Road, Cambridge CB2 1EW

Supplementary Material

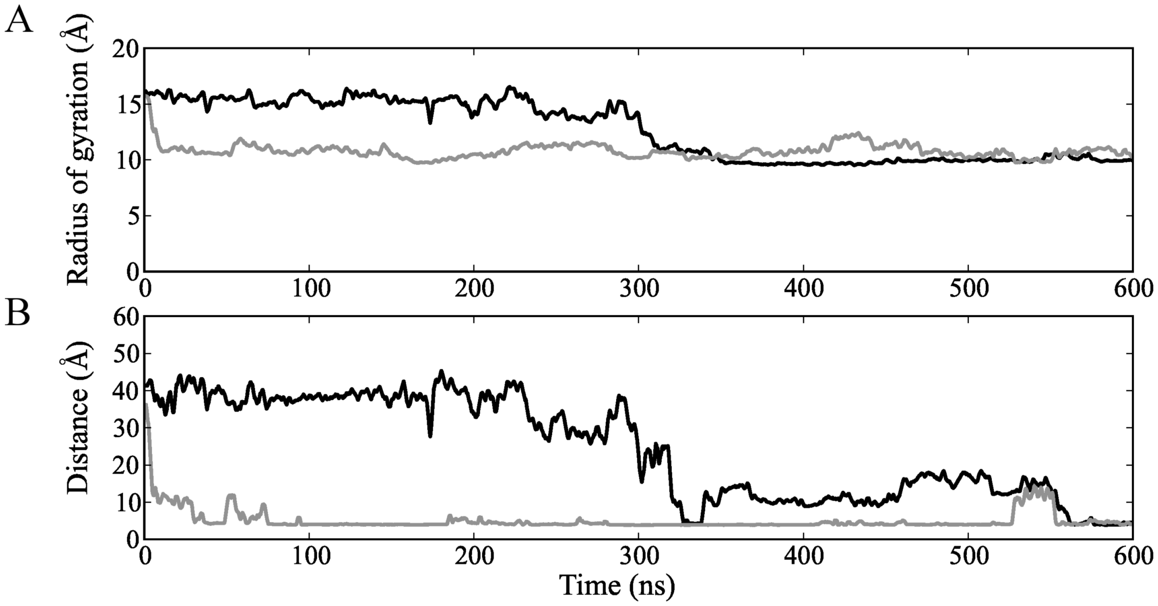

FIG. 1: Time evolution of (A) the radius of gyration and (B) distances between E129-R155 and E130-R155 during the molecular dynamics simulations at 280 and 300 K (black and grey lines respectively).

*Electronic address: djw34@cam.ac.uk

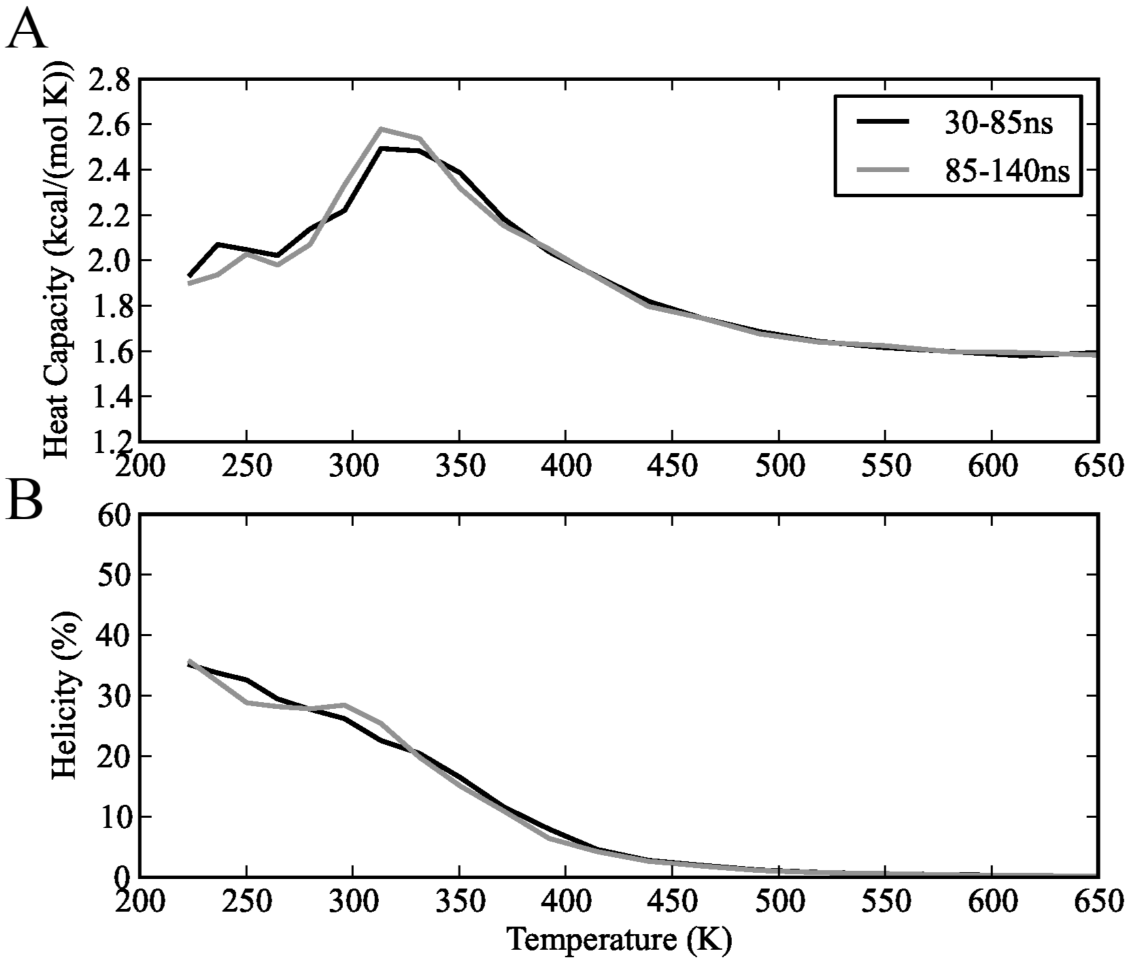

FIG. 2: REMD simulations of the PUMA helical peptide. (A) Heat capacities in kcal/(mol K) and (B) residual helicity percentages are represented between 30-8 5ns (black lines) and 85-140 ns (grey lines).

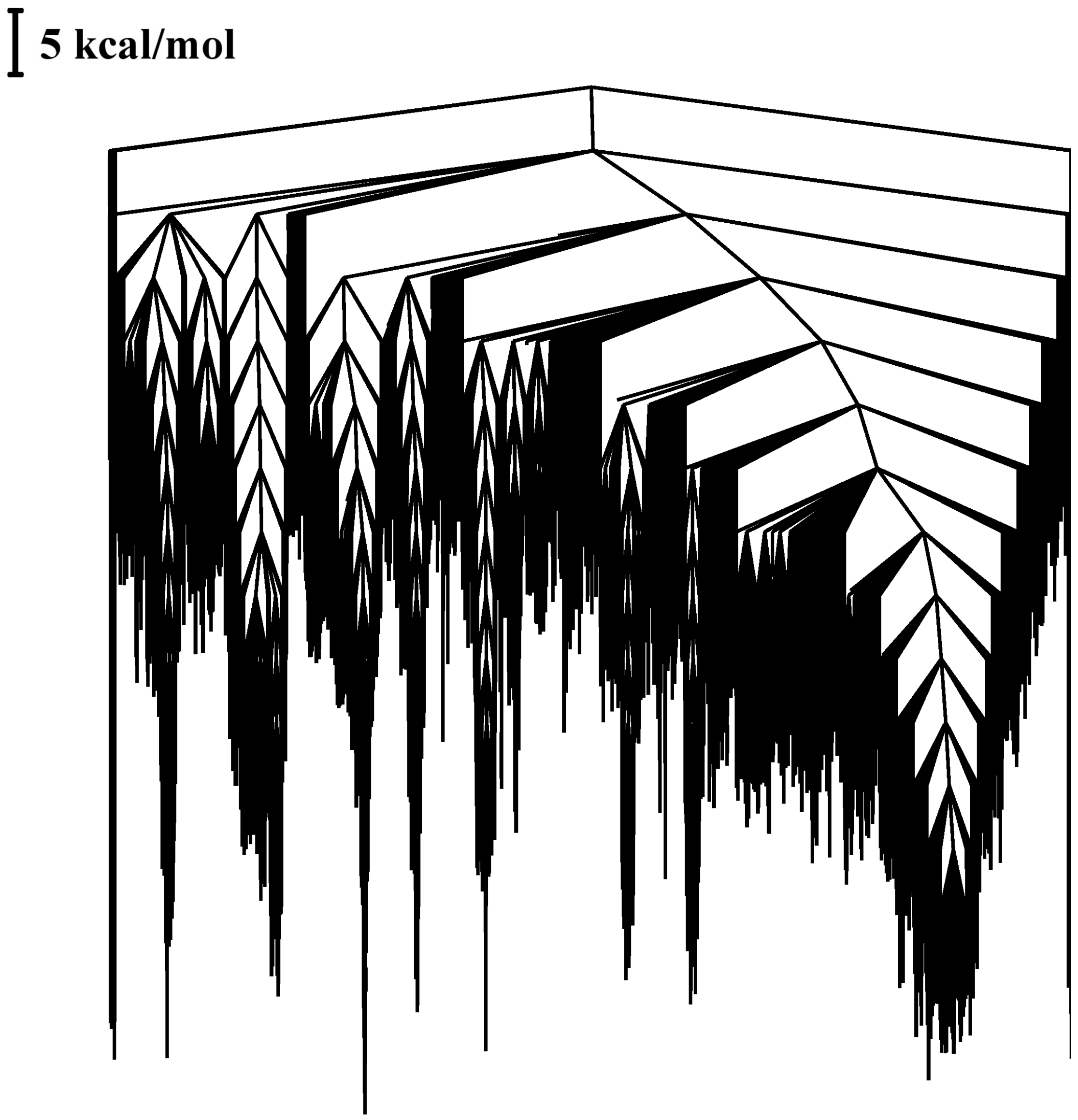

FIG. 3: Free energy disconnectivity graph constructed from the most populated structures obtained in the REMD simulations at 280 K.

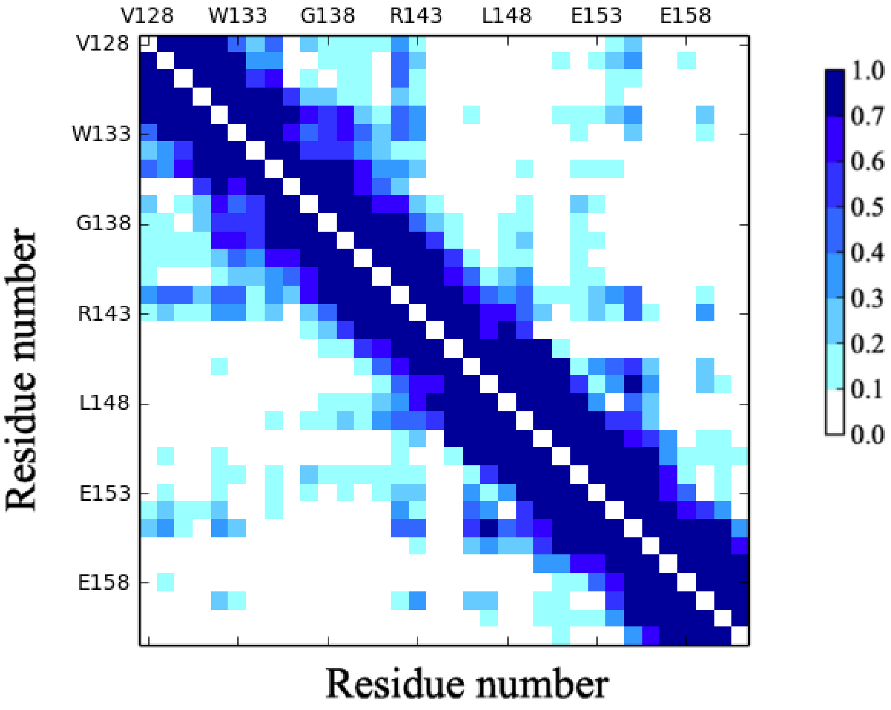

FIG. 4: Residue contact map calculated at 280 K.

Conformation A	Conformation B									
	1	2	3	4	5	6	7	8	9	10
1		658.4	$0.11 \mathrm{x} 10^{-33}$	$0.9 \mathrm{x} 10^{-31}$	$0.11 \mathrm{x} 10^{-37}$	$0.66 \mathrm{x} 10^{-37}$	$0.25 \mathrm{x} 10^{-23}$	$0.54 \mathrm{x} 10^{-32}$	$0.13 \mathrm{x} 10^{-31}$	$0.4 \mathrm{x} 10^{-27}$
2	$5 \mathrm{x} 10^{6}$		$0.9 \mathrm{x} 10^{-30}$	$0.74 \mathrm{x} 10^{-27}$	$0.9 \mathrm{x} 10^{-34}$	$0.55 \mathrm{x} 10^{-33}$	$0.21 \mathrm{x} 10^{-19}$	$0.44 \mathrm{x} 10^{-28}$	$0.1 \mathrm{x} 10^{-27}$	$0.33 \mathrm{x} 10^{-23}$
3	$0.34 \mathrm{x} 10^{-37}$	$0.34 \mathrm{x} 10^{-37}$		$0.34 \mathrm{x} 10^{-37}$	$0.34 \mathrm{x} 10^{-41}$	$0.21 \mathrm{x} 10^{-40}$	$0.34 \mathrm{x} 10^{-37}$	$0.33 \mathrm{x} 10^{-37}$	$0.34 \mathrm{x} 10^{-37}$	$0.34 \mathrm{x} 10^{-37}$
4	$0.13 \mathrm{x} 10^{-30}$	$0.13 \mathrm{x} 10^{-30}$	$0.16 \mathrm{x} 10^{-33}$		$0.16 \mathrm{x} 10^{-37}$	$0.95 \mathrm{x} 10^{-37}$	$0.13 \mathrm{x} 10^{-30}$	$0.73 \mathrm{x} 10^{-32}$	$0.16 \mathrm{x} 10^{-31}$	$0.13 \mathrm{x} 10^{-30}$
5	$0.16 \mathrm{x} 10^{-32}$	$0.16 \mathrm{x} 10^{-32}$	$0.16 \mathrm{x} 10^{-32}$	$0.16 \mathrm{x} 10^{-32}$		$0.14 \mathrm{x} 10^{-32}$	$0.16 \mathrm{x} 10^{-32}$	$0.16 \mathrm{x} 10^{-32}$	$0.16 \mathrm{x} 10^{-32}$	$0.16 \mathrm{x} 10^{-32}$
6	$0.36 \mathrm{x} 10^{-37}$	$0.36 \mathrm{x} 10^{-37}$	$0.36 \mathrm{x} 10^{-37}$	$0.36 \mathrm{x} 10^{-37}$	$0.52 \mathrm{x} 10^{-38}$		$0.36 \mathrm{x} 10^{-37}$	$0.36 \mathrm{x} 10^{-37}$	$0.36 \mathrm{x} 10^{-37}$	$0.37 \mathrm{x} 10^{-37}$
7	$0.35 \mathrm{x} 10^{-19}$	$0.35 \mathrm{x} 10^{-19}$	$0.15 \mathrm{x} 10^{-29}$	$0.13 \mathrm{x} 10^{-26}$	$0.16 \mathrm{x} 10^{-33}$	$0.94 \mathrm{x} 10^{-33}$		$0.76 \mathrm{x} 10^{-28}$	$0.18 \mathrm{x} 10^{-27}$	$0.57 \mathrm{x} 10^{-23}$
8	$0.13 \mathrm{x} 10^{-29}$	$0.13 \mathrm{x} 10^{-29}$	$0.27 \mathrm{x} 10^{-31}$	$0.13 \mathrm{x} 10^{-29}$	$0.27 \mathrm{x} 10^{-35}$	$0.16 \mathrm{x} 10^{-34}$	$0.14 \mathrm{x} 10^{-29}$		$0.54 \mathrm{x} 10^{-30}$	$0.13 \mathrm{x} 10^{-29}$
9	$0.26 \mathrm{x} 10^{-18}$	$0.26 \mathrm{x} 10^{-18}$	$0.23 \mathrm{x} 10^{-20}$	$0.23 \mathrm{x} 10^{-18}$	$0.23 \mathrm{x} 10^{-24}$	$0.14 \mathrm{x} 10^{-23}$	$0.26 \mathrm{x} 10^{-18}$	$0.8 \mathrm{x} 10^{-19}$		$0.26 \mathrm{x} 10^{-18}$
10	$0.43 \mathrm{x} 10^{-22}$	$0.43 \mathrm{x} 10^{-22}$	$0.12 \mathrm{x} 10^{-28}$	$0.95 \mathrm{x} 10^{-26}$	$0.12 \mathrm{x} 10^{-32}$	$0.7 \mathrm{x} 10^{-32}$	$0.43 \mathrm{x} 10^{-22}$	$0.57 \mathrm{x} 10^{-27}$	$0.13 \mathrm{x} 10^{-26}$	

TABLE I: Supplementary table 1: Estimated rate contants (in s^{-1}) at 280 K between the 10 most populated structures from the REMD simulations. The values in the upper triangular part of the table correspond to the rates between conformation A and B, $\mathrm{k}_{B \leftarrow A}$ and in the lower part between conformation B and A, $\mathrm{k}_{A \leftarrow B}$.
